# Preclinical safety and immunogenicity of *Streptococcus pyogenes* (Strep A) peptide vaccines

**DOI:** 10.1038/s41598-020-80508-6

**Published:** 2021-01-08

**Authors:** Simone Reynolds, Manisha Pandey, Jessica Dooley, Ainslie Calcutt, Michael Batzloff, Victoria Ozberk, Jamie-Lee Mills, Michael Good

**Affiliations:** Institute for Glycomics, Griffith University, Gold Coast, Australia

**Keywords:** Toxicology, Medical research, Preclinical research, Vaccines

## Abstract

We have developed two candidate vaccines to protect against multiple strains of Strep A infections. The candidates are combinatorial synthetic peptide vaccines composed of a M protein epitope (J8 or p*17) and a non-M protein epitope (K4S2). To enhance immunogenicity, each peptide is conjugated to the carrier protein CRM_197_ (CRM) and formulated with aluminium hydroxide adjuvant Alhydrogel (Alum) to make the final vaccines, J8-CRM + K4S2-CRM/Alum and p*17-CRM + K4S2-CRM/Alum. The safety and toxicity of each vaccine was assessed. Sprague Dawley rats were administered three intramuscular doses, over a six-week study with a 4-week recovery period. A control group received CRM only formulated with Alum (CRM/Alum). There was no evidence of systemic toxicity in the rats administered either vaccine. There was an associated increase in white blood cell, lymphocyte and monocyte counts, increased adrenal gland weights, adrenocortical hypertrophy, and increased severity of granulomatous inflammation at the sites of injection and the associated inguinal lymph nodes. These changes were considered non-adverse. All rats administered vaccine developed a robust and sustained immunological response. The absence of clinical toxicity and the development of an immunological response in the rats suggests that the vaccines are safe for use in a phase 1 clinical trial in healthy humans.

## Introduction

Infection with *Streptococcus pyogenes* (group A streptococcus, Strep A) is responsible for a large number of clinical manifestations. It is a major problem in many developing countries and Indigenous populations of developed countries where poor access to health care, socioeconomic challenges and disadvantage exist. Pathology due to Strep A can be divided into acute suppuration (pus forming) and post-streptococcal sequelae. The former include the relatively benign streptococcal pharyngitis and pyoderma, particularly common in children, and the far more serious necrotizing fasciitis, pneumonia, and toxic shock-like syndrome, primarily affecting older individuals. The post-streptococcal sequelae include rheumatic fever (RF), rheumatic heart disease (RHD), RHD-associated stroke and acute post-streptococcal glomerulonephritis (APSGN). RHD, which is associated with an autoimmune response, is of most concern, as it can lead to heart failure and a significantly shortened lifespan.

Vaccination is one of the most effective preventative methods for controlling infectious diseases. Substantial progress has been made with a recombinant multi-valent protein vaccine that consists of multiple N-terminal epitopes of the M-protein from different Strep A strains (*emm* types) isolated mainly in the USA^1,2^. These studies have demonstrated that this approach induces antibodies capable of recognizing strains specific to the *emm*-type protein represented in the vaccine, and sub *emm*-type variants that differ slightly in the amino acid sequence from those in the vaccine construct^3,4^. However, the specificity of the induced antibodies limits the use of this vaccine in developing countries where the prevalence of Strep A infection is very high and there is a rapid turnover of *emm*-types that significantly differ from those found in the developed countries such as USA. This limitation was highlighted within the Phase 1 study, when participant antibodies demonstrated a mean response rate of 38% to the 31 vaccine peptides^4^.

Epitope(s) based on the highly conserved C-repeat region of the M-protein are promising candidates for vaccine development as this region of the M-protein is conserved across Strep A strains. Epitopes in this region contribute to immunity against homologous and heterologous strains in animals following vaccination and are recognized by the sera of most adults living in areas of Strep A endemicity^5,6^.

In addition to exploring a vaccine targeting the M-protein, there is a body of research investigating the utility of a vaccine that targets non-M proteins of Strep A. This work focuses primarily on the Strep A virulence factors that are upregulated in Strep A mutant strains, referred to as CovR/S mutants. Strep A CovR/S mutants display very high virulence with respect to invasive disease. There are several virulence factors under investigation including SpeA, SpeB, SpeC, and Serum Opacity Factor^7^ and those contained in the candidate vaccine Combo5 (arginine deiminase, C5a protease, streptolysin O, SpyCEP and trigger factor)^8^. Antigens from many of these virulence proteins have a global coverage or carriage of over 99% with the added feature of low allelic variation (< 2%) making them highly suitable target antigens for vaccine development^9^. One of the major virulence proteins upregulated in CovR/S mutation is *S. pyogenes* cell envelope protease (SpyCEP). SpyCEP cleaves human interleukin 8 (IL-8) and disrupts neutrophil chemotaxis to the site of infection. Antibodies induced against SpyCEP have been shown to protect human IL-8 degradation and enhance vaccine efficacy against CovR/S mutants^10,11^.

The approach we have taken is to combine a modified peptide derived from the C-repeat region of the M-protein^5,10,12–14^ and an epitope of SpyCEP; the M protein peptides J8^5,15^ and p*17^16^ and the non-M protein peptide K4S2^17^. The strategy of using a conserved Strep A epitope circumvents the strain diversity and cross reactivity limitations of the hypervariable amino terminal-based vaccines. It has the potential advantage of inducing protection against infections caused by different Strep A *emm* types worldwide, particularly those in less developed countries where the turnover of Strep A strains is very rapid. The inclusion of peptide K4S2 induces antibodies that protect host IL-8 from SpyCEP mediated proteolysis, thus enabling neutrophil recruitment to the infection site^17^.

Here, we report a preclinical toxicity and immunogenicity study in rats, of the lead candidate vaccines, J8-CRM + K4S2-CRM/Alum and p*17-CRM + K4S2-CRM/Alum. We also provide supporting evidence of the protective efficacy of the combination vaccines in murine models of Strep A skin and systemic infection. In murine studies, p*17 and K4S2 were conjugated to diphtheria toxoid (DT) and J8 and K4S2 were conjugated to CRM, an enzymatically inactive non-toxic form of diphtheria toxin. The data demonstrate that these vaccine formulations are efficacious against infections induced by both CovR/S wild type (WT) and mutant strain (MT) organisms in outbred and inbred models^10,17–19^.

## Results

### Vaccines are immunogenic and protective in inbred and outbred mice

**Figure 1:**
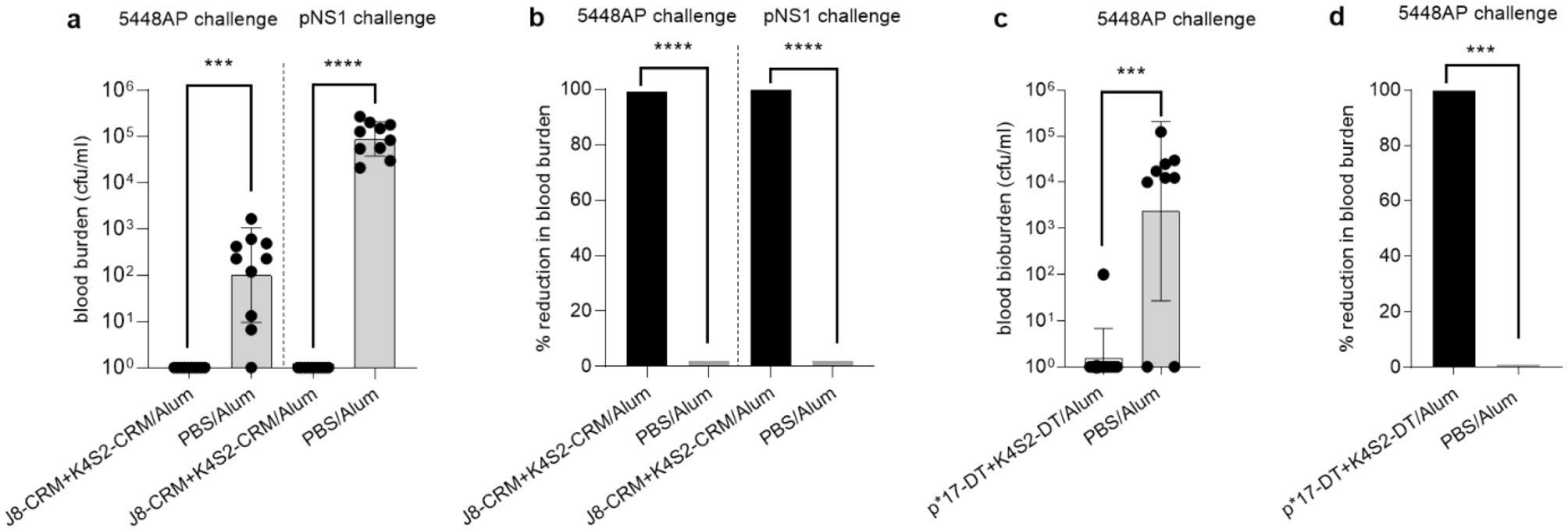
Protective efficacy of J8-CRM + K4S2-CRM/Alum against invasive disease following skin challenge. (**a**,**b**) Cohorts of SWISS mice (n = 10) were immunized intramuscularly with 0.025 mg/dose of J8-CRM + K4S2-CRM/Alum on days 1, 22 and 43. Post final boost, mice were challenged via skin with a Strep A mutant 5448AP or wild type strain pNS1. Mice were culled on day 7 post challenge and blood samples were plated to determine bacterial load. (**c**,**d**) Protective efficacy of p*17-DT + K4S2-DT/alum against invasive disease following skin challenge of BALB/c mice. BALB/c mice (n = 10/group, female, 4–6 weeks old) were immunised intramuscularly with 0.025 mg/dose of p*17-DT + K4S2-DT/Alum on days 0, 21 and 42. Two weeks post final immunization, mice were challenged via the skin with 5448AP. Mice were culled on day 7 post challenge and blood samples were plated to determine bacterial load (colony forming units [cfu]). Individual blood bacterial burden (cfu/mL) (**a** and **c**) and group percentage reduction in blood bacterial burden (**b** and **d**) are shown. Protection is defined as percent reduction in bacterial burden and is calculated using the Geomean bacterial load in blood of vaccinated and control group (PBS/Alum). Statistical comparisons and graphs generated using Mann–Whitney test were performed using GraphPad Prism (8.1.2). ***p < 0.001, ****p < 0.0001*.*

The immunogenicity and protective efficacy of combination vaccines against CovR/S WT and MT strains were assessed in the murine model of Strep A skin infection, in outbred and inbred mice. SWISS outbred mice (n = 10) were administered J8-CRM + K4S2-CRM/Alum and BALB/c mice (n = 10) were administered p*17-DT + K4S2-DT/Alum, as three intramuscular injections. Final immunizations were followed by skin challenges with Strep A CovR/S MT 5448AP (*emm* 1) or WT (pNS1, *emm* 100) strain. Both J8-CRM + K4S2-CRM/Alum and p*17-DT + K4S2-DT/Alum induced peptide specific IgG titers between 10^4^–10^6^ (data not shown) and were efficacious in protecting mice (> 99% reduction in bacterial load) against Strep A induced systemic infection from either the WT or MT Strep A strain (Fig. 1a–d).

### A repeat-dose toxicity study in Sprague Dawley rats of two candidate vaccines

Two candidate vaccines, J8-CRM + K4S2-CRM/Alum and p*17-CRM + K4S2-CRM/Alum, and a control article, CRM/Alum, were administered to male and female Sprague–Dawley rats (n = 20, 10/sex/group) on days 1, 22 and 43. Rats received 0.5 mL intramuscular injections (0.25 mL/thigh) as follows: Group 1—0.065 mg CRM/Alum (control); Group 2—0.1 mg J8-CRM + K4S2-CRM/Alum; Group 3—0.1 mg p*17-CRM + K4S2-CRM/Alum*.* The control article, CRM/Alum, contained 0.065 mg of CRM which is the average concentration of CRM contained in either vaccine. J8-CRM + K4S2-CRM/Alum contained 0.05 mg of J8-CRM and 0.05 mg of K4S2-CRM for a net peptide conjugate concentration of 0.1 mg. p*17-CRM + K4S2-CRM/Alum contained 0.05 mg of p*17-CRM and 0.05 mg of K4S2-CRM for a net peptide conjugate concentration of 0.1 mg (Table 1). All study animals were observed throughout treatment until half of the study animals were euthanized and necropsied on study day 57 (Main cohort, 2 weeks after dose 3); the remaining rats were euthanized and necropsied on study day 86 (Recovery cohort, 6 weeks after dose 3).

**Table 1:** Toxicity study groups and schedule.

Group	Immunization			Necropsy	
	#1	#2	#3	Main	Recovery
	Day 1	Day 22–23	Day 43	Day 57	Day 86
1. CRM/Alum	0.065 mg/dose	0.065 mg/dose	0.065 mg/dose	5 M,5F	5 M,5F
2. J8-CRM + K4S2-CRM/Alum	0.1 mg/dose	0.1 mg/dose	0.1 mg/dose	5 M,5F	5 M,5F
3. p*17-CRM + K4S2-CRM/Alum	0.1 mg/dose	0.1 mg/dose	0.1 mg/dose	5 M,5F	5 M,5F

Alum, aluminum hydroxide adjuvant; CRM, cross-reacting material 197; M, male; F, female.

All animals survived until scheduled sacrifice. No adverse vaccine-related findings were reported in the clinical signs, body weights, food consumption, ophthalmology, gross pathology or macroscopic observations. All microscopic changes observed in vaccine groups were within the spectrum of expected findings post-vaccination and considered non-adverse.

### Local irritation assessment (draize scoring)

Minor erythema and edema were observed in all dose groups, after the first and second doses. Following the third dose, an effect on erythema and edema at the injection sites were observed in the female vaccine groups when compared to the females in the CRM/Alum control group. Findings in the male vaccine groups scores compared to the control group were not considered toxicologically relevant.

**Figure 2:**
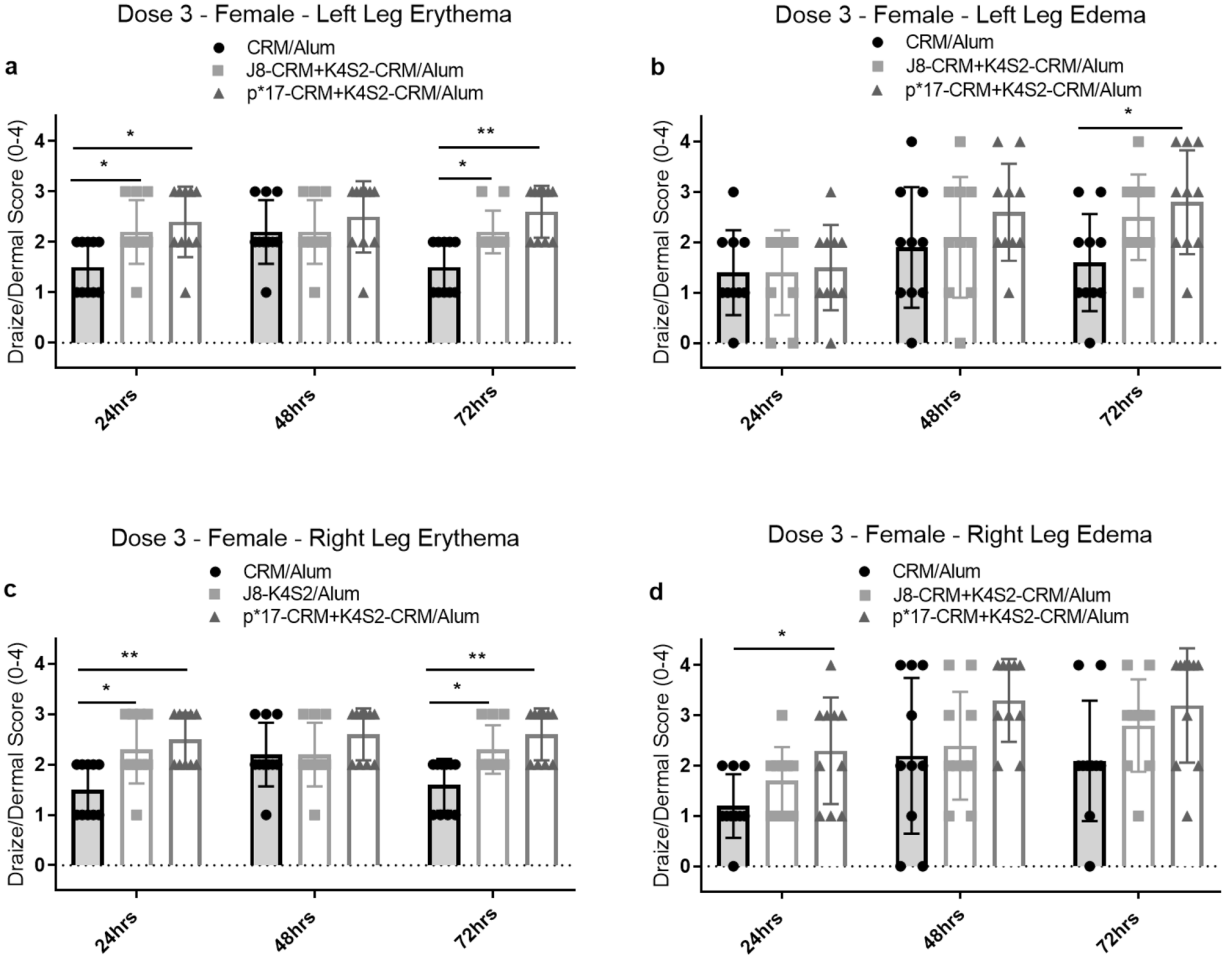
Female Draize scoring after third dose (day 43). Draize scores are treated as replicates (n = 10), for each clinical observation, at a given time point. All four clinical observations; (**a**) left leg erythema; (**b**) left leg edema; (**c**) right leg erythema and (**d**) right leg edema are shown for each time point. Data for an observation, at a given time point, is displayed as group score mean with SD. Statistical analysis and graphs generated using T test to compare each vaccine group to the CRM/Alum control, at each time point, were performed using GraphPad Prism (8.1.2). *p < 0.05, **p < 0.01*.*

In the female control group, the onset of erythema occurred 48 h post dose and began to resolve 72 h post dose. In both the female vaccine groups, the peak onset of erythema occurred at 24 h and persisted through to 72 h post dose. A significant increase in erythema (left and right) was observed at 24 and 72 h post dose in the both the vaccine groups. Additionally, at 72 h post dose, an increase in left and right leg edema scores was observed in the female J8-CRM + K4S2-CRM/Alum group, compared to the control group. This was also observed in the female p*17-CRM + K4S2-CRM/Alum group, where a significant increase in left leg edema and an increase (not significant) in right leg edema was observed at 72 h post dose (Fig. 2). These observations were considered non-adverse and no other toxicologically relevant effects on local irritation were noted.

### Clinical pathology

No adverse vaccine-related findings in the clinical chemistry, coagulation or urinalysis were reported throughout the study phases. No effect on hematology parameters were considered vaccine-related, during the dosing phase of the study. At the day-86 sacrifice, a modest increase in white blood cell and lymphocyte counts were reported in both female groups receiving vaccine (5% significant difference from CRM/Alum control group). Mildly elevated monocyte counts were also observed at day 86 in females receiving p*17-CRM + K4S2-CRM/Alum (5% significant difference from CRM/Alum control group). These increases were not considered adverse, but rather a reflection of the immune response induced by immunization.

### Immunogenicity and functional assessment of vaccines in rats

**Figure 3:**
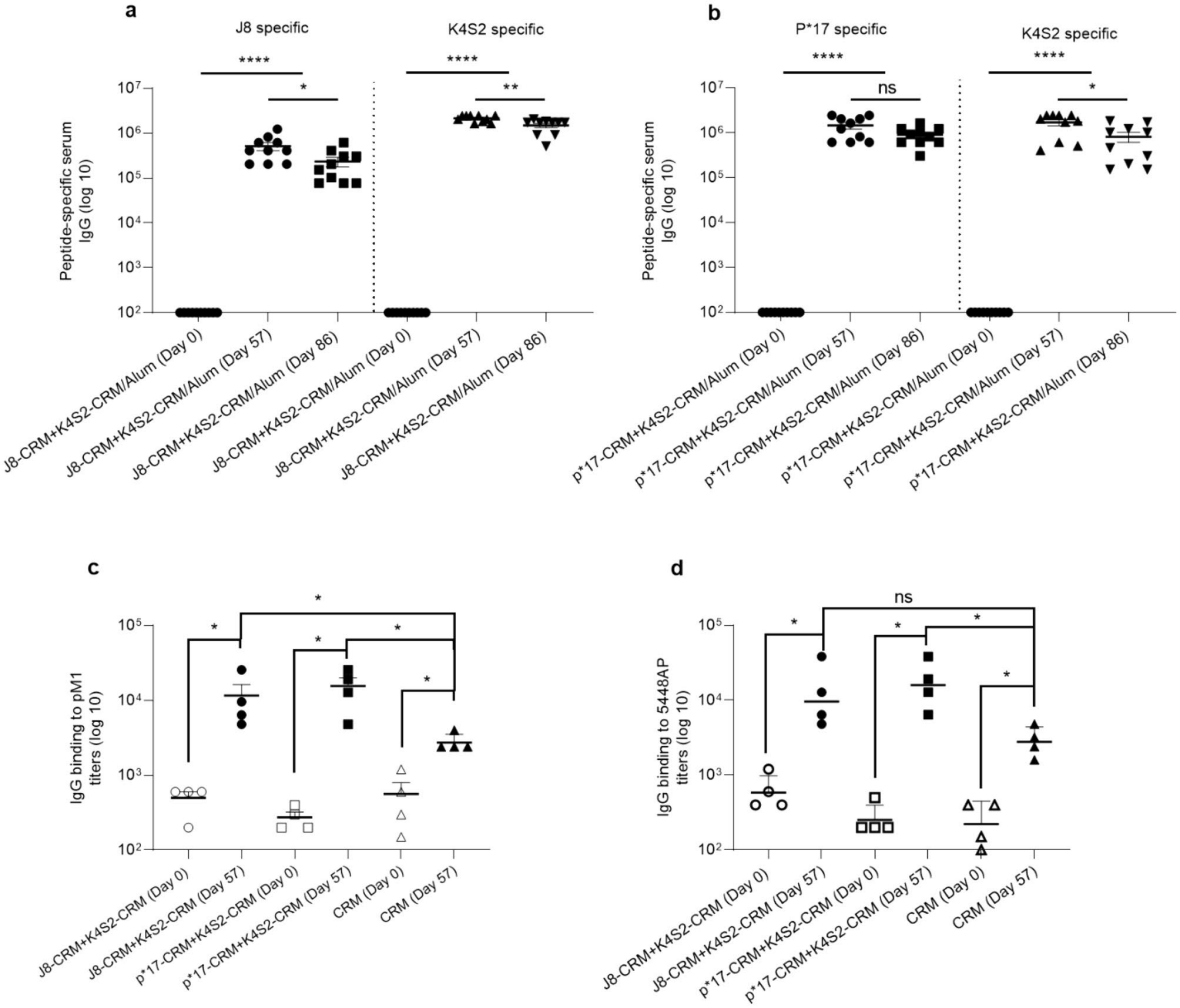
Vaccine immunogenicity and functionality of vaccine-induced antibodies. (**a**,**b)** Vaccine peptide specific serum IgG titers induced in Sprague–Dawley rats after vaccination with J8-CRM + K4S2-CRM/Alum or p*17-CRM + K4S2-CRM/Alum. Sprague–Dawley rats were immunized intramuscularly with 0.1 mg/dose of J8-CRM + K4S2-CRM/Alum or p*17-CRM + K4S2-CRM/Alum on days 0, 21 and 42. Rats were euthanized at day 57 and day 86. J8, p*17 and K4S2 specific serum IgG titers (Geomean) are shown for individual rats vaccinated with J8-CRM + K4S2-CRM/Alum (**a**) or p*17-CRM + K4S2-CRM/Alum (**b**) euthanized at day 57 (n = 10; 5 male, 5 female) or 86 (n = 10; 5 male, 5 female). Pre-vaccination (day 0) serum IgG titers of the same rats assessed at day-57 or -87 are also shown. Samples were considered positive when the mean value of the absorbance, of the highest dilution (1:100), was > 3SD above the mean OD of the negative control. (**c**,**d**) Strep A surface binding of vaccine induced antibodies. Sera collected from rats vaccinated with J8-CRM + K4S2-CRM/Alum, p*17-CRM + K4S2-CRM/Alum or CRM/Alum in the toxicology study was assessed for direct IgG binding to heat killed pM1 (**c**) and 5448AP (**d**). Mean individual titers of sera collected from days-0 (n = 4) and -57 (n = 4) and assayed by ELISA are shown. Statistical analysis and graphs generated using a Mann–Whitney test to compare all groups, were performed using GraphPad Prism (8.1.2) *p < 0.05, **p < 0.01, ***p < 0.001, ****p < 0.0001.

There were no peptide-specific antibodies detected in sera samples collected from rats prior to dosing (Fig. 3a,b). There was a robust immune response to the Strep A peptides, J8 and K4S2, in the J8-CRM + K4S2-CRM/Alum main group animals (day 57 sera) that persisted in the recovery group animals (day 86 sera) (Fig. 3a). Additionally, there was a robust immune response to the Strep A peptides, p*17 and K4S2, in the p*17-CRM + K4S2-CRM/Alum main group animals that persisted in the recovery animals (Fig. 3b).

The functionality of vaccine-induced antibodies in rats was assessed via antibody recognition of bacterial proteins. Vaccine induced antibodies generated in the toxicology study were assessed for direct recognition of bacteria proteins by ELISA. Sera collected pre-dosing (day-0) and at main necropsy (day-57), from each cohort; J8-CRM + K4S2-CRM/Alum, p*17-CRM + K4S2-CRM/Alum and CRM/Alum (n = 4/cohort), were added to wells coated with heat killed, whole cell preparations of Strep A *emm* 1 strains pM1 (WT) and 5448AP (MT). Direct binding to bacteria proteins was defined by total IgG titers. Overall, significant binding to bacteria proteins was observed in the day-57 titers for all cohorts compared to the day 0 titers. A comparative analysis between the binding of candidate vaccine-induced antibodies and the control CRM/Alum-induced antibodies was also made. No significant difference in the binding capacity of day-57 J8-CRM + K4S2-CRM/Alum and p*17-CRM + K4S2-CRM/Alum-induced antibodies to wild type or mutant bacteria was observed. Binding to WT pM1 by either J8-CRM + K4S2-CRM/Alum or p*17-CRM + K4S2-CRM/Alum vaccine-induced antibodies was significantly higher (*p* < 0.05) than the CRM/Alum-induced antibodies. Binding to MT 5448AP by p*17-CRM + K4S2-CRM/Alum vaccine-induced antibodies was significantly higher (*p* < 0.05) than the CRM/Alum-induced antibodies. There was no significant difference in binding to 5448AP between day-57 J8-CRM + K4S2-CRM/Alum and CRM/Alum. Finally, no significant difference in binding was observed between day 0 titers for all cohorts (Fig. 3c,d).

### Organ weights

No vaccine-related weight changes were reported in the brain, epididymis, heart, kidney, liver or lungs at the day-57 or -86 necropsies. A minimal or mild increase in the adrenal gland weights in male rats receiving the vaccine candidates, compared to the CRM/Alum control, was observed at the day-57 sacrifice. This was considered a common change in toxicologic pathology. The histopathologic correlate was adrenocortical hypertrophy, which is typically secondary to physiologic stress. No vaccine-related organ weight changes were reported at the day-86 sacrifice.

### Histopathological analysis

A list of the organs and tissues retained for histopathological examination is provided in Supplementary Table S1. In summary, microscopic observations in the rats that received vaccine, when compared to the CRM/Alum control rats, were granulomatous inflammation at the right and/or left injection sites and inguinal lymph nodes in males and/or females, and adrenocortical hypertrophy in males. All observations were considered non-adverse.

At day-57 sacrifice, the overall incidence of granulomatous inflammation observed at the right and left injection sites was comparable between the control group and the vaccine groups (Supplementary Table S2). There was a slight increase in the severity of the granulomatous inflammation in both the left and right injection sites of male rats and the right injection site of female rats that received J8-CRM + K4S2-CRM/Alum. Likewise, there was a small increase in severity of the granulomatous inflammation in the left and right injection sites of female rats and the left injection site of male rats that received p*17-CRM + K4S2-CRM/Alum. At day 86, a slight increase in severity of granulomatous inflammation persisted in the right injection site of one of five female rats that received p*17-CRM + K4S2-CRM/Alum. No evidence of persisting granulomatous inflammation was observed in the left injection site. The injection site granulomas contained aggregates of macrophages interspersed with small numbers of lymphocytes and plasma cells typically around the periphery of the macrophage aggregates.

An increase in the severity of granulomatous inflammation in vaccine groups was observed in the inguinal lymph nodes (Supplementary Table S3). At the day-57 sacrifice, the group administered J8-CRM + K4S2-CRM/Alum experienced a slight increase in the severity of granulomatous inflammation of the left and right nodes of female rats and the right nodes of male rats. The overall incidence of this finding was considered similar across the control and vaccine groups. In addition, at the day-86 sacrifice, a small increase in severity was observed in the left inguinal lymph nodes of female rats in both vaccine groups. The increased granulomatous inflammation was not observed in the left inguinal nodes of male rats or the right inguinal nodes of any of the animals. As with the granulomatous inflammation at the injection site, the granulomas in the inguinal lymph nodes, were characterized by the presence of aggregates of macrophages. The cytoplasmic material of the individual macrophages was suggestive of phagocytosed adjuvant.

At the day-57 sacrifice, minimal or mild adrenocortical hypertrophy was reported in male rats in both vaccine groups, compared to the CRM/Alum control group (Supplementary Table S4). No incidence of moderate or marked hypertrophy was seen in any animal. The cytoplasm of the cortical epithelial cells was described as homogenously eosinophilic, whilst the increase in cell size resulted in compression of the proximate blood vessels. The adrenocortical hypertrophy was the histopathologic correlate for the increased adrenal gland weights, a typical stress response observed in toxicity studies^20^. In the context of this study, the observation was considered secondary to physiologic stress and not vaccine-related. There was no report of adrenocortical hypertrophy at the day-86 sacrifice.

### Examination of the kidneys, brain and joints

No vaccine-related adverse findings were reported in the kidneys. At both the day-57 and -86 necropsies, there was no evidence of macroscopic damage (lesions) or changes to the organ weight. There was also no effect on urinalysis parameters, including blood and protein, observed after the administration of either vaccine compared to the CRM/Alum control.

A broad spectrum of histopathological findings in the kidneys were reported at both day-57 and -86 necropsies and are outlined in Table 2. These findings were of similar incidence and severity in the CRM/Alum control and vaccine treated groups. The observations were considered to be incidental for the strain and age of the rats and not vaccine-related.

**Table 2:** Kidneys—histopathology observations.

	Males (5 per group)			Females (5 per group)		
	CRM	J8-K4S2	p*17-K4S2	CRM	J8-K4S2	p*17-K4S2
**Day 57**						
Within normal limits	2	3	4	5	4	3
Chronic progressive nephropathy, minimal	3	2	0	0	1	0
Dilation; tubular, minimal	0	0	1	0	0	1
Atrophy; tubular, minimal	0	0	1	0	0	0
Infiltration; mixed, minimal	0	0	0	0	0	1
**Day 86**						
Within normal limits	2	1	4	3	4	2
Cast	1	1	0	1	0	1
Chronic progressive nephropathy, minimal	0	1	1	0	1	1
Chronic progressive nephropathy, mild	1	0	0	0	0	0
Cyst; tubular	1	1	0	0	0	0
Dilation; pelvic, mild	1	0	0	0	0	0
Infiltration, lymphocytic; interstitial	0	1	0	1	0	1
Hypertrophy; tubular, minimal	0	0	0	0	0	1

CRM, CRM/Alum; J8-K4S2, J8-CRM + K4S2-CRM/Alum; p*17-K4S2; p*17-CRM + K4S2-CRM/Alum.

An examination of the brains of all animals found no evidence of macroscopic damage (lesions) or histopathological findings at the day-57 necropsy and no evidence of a change to the organ weight at day 57 or 86. At day 86, a single male rat administered p*17-CRM + K4S2-CRM/Alum was found to have mild dilation of a ventricle. No other histological findings related to the brain were reported for day 86.

There was also no report of any inflammation observed in the joints of any animals receiving vaccine.

### Examination of the heart

A microscopic examination of the heart found no evidence of valvulitis, however, minimal cardiomyopathy was observed in 11 of 60 animals and mild cardiomyopathy in 1 animal; no incidence of moderate or marked myopathy was seen in any animal (Table 3). Statistical analysis (Fisher’s exact test, *p* < 0.05, GraphPad Prism) of cardiomyopathy observed at day 56 in groups that received vaccine compared to the control article determined no significant difference in the rates observed (J8-CRM + K4S2-CRM/Alum; *p* value 0.9999, p*17-CRM + K4S2-CRM/Alum; *P* value 0.9999). In addition, observations at day 86 in groups that received vaccine compared to control article determined no significant difference in the cardiomyopathy rates observed (J8-CRM + K4S2-CRM/Alum; *p* value 0.0867, p*17-CRM + K4S2-CRM/Alum; *p* value 0.2105). The observation of cardiomyopathy is in-line with other toxicology studies, where spontaneous cardiomyopathy in young Sprague–Dawley rats is a common background finding and has been reported to have up to a 100% incidence, particularly in males^21^. The overall conclusion was that it is an incidental finding not related to the vaccines. This supports our previous findings with J8 in a heart valvulitis model^13^.

**Table 3:** Observed cardiomyopathy.

Main necropsy	Males (5 per group)			Females (5 per group)		
	CRM	J8-K4S2	p*17-K4S2	CRM	J8-K4S2	p*17-K4S2
**Day 57**						
Minimal	1	2	2	0	0	0
Mild	0	0	0	0	0	0

Recovery necropsy	Males (5 per group)			Females (5 per group)		
	CRM	J8-K4S2	p*17-K4S2	CRM	J8-K4S2	p*17-K4S2
**Day 86**						
Minimal	0	2	2	0	1	1
Mild	0	1	0	0	0	0

### Stability of vaccines over 12 months

**Figure 4:**
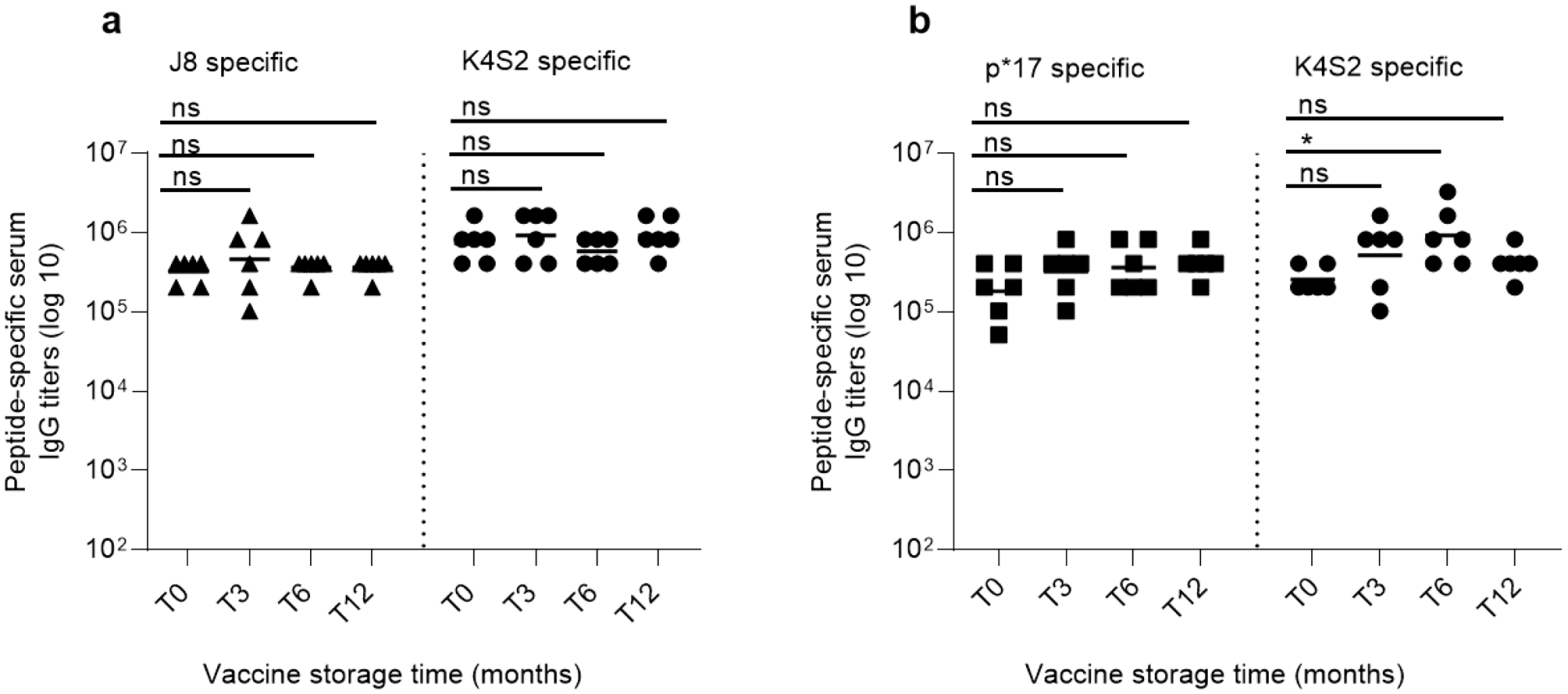
Stability of vaccines. Peptide specific serum IgG titers induced in Sprague–Dawley rats, after vaccination with J8-CRM + K4S2-CRM/Alum or p*17-CRM + K4S2-CRM/Alum stored for 0, 3, 6 and 12 months, were compared. Sprague–Dawley rats were immunized once intramuscularly with 0.1 mg/dose of J8-CRM + K4S2-CRM/Alum or p*17-CRM + K4S2-CRM/Alum. 18 ± 2 days following immunization, rats were bled. J8, p*17and K4S2 specific serum IgG titers are shown. IgG titers in rats (n = 6; 3 male, 3 female) vaccinated with J8-CRM + K4S2-CRM/Alum (**a**) or p*17-CRM + K4S2-CRM/Alum (**b**) stored for 0 (T0), 3 (T3), 6 (T6) and 12 months (T12). Samples were considered positive when the mean value of the absorbance, of the highest dilution (1:100) was 3SD above the mean OD of the negative control. Statistical analysis using a Mann–Whitney test to compare all groups to the T0 group, were performed using GraphPad Prism (8.1.2). *p < 0.05.

Stability of the vaccines, for long-term storage at 5 °C ± 3 °C, was assessed at 3-, 6-, and 12-month time points (T3, T6, T12), post manufacturing (Time 0). Stability parameters assessed were appearance, pH, adsorption to Alum (Table 4) and immunogenicity (Fig. 4). The vaccines were shown to be stable for up to 12 months, when stored at 5 °C ± 3 °C, with no significant changes noted to any parameters measured over that time. This included inducing an expected potent immune response in Sprague Dawley rats.

**Table 4:** Stability result summary.

	Specification	T0 months	T3 months	T6 months	T12 months
**J8-CRM + K4S2-CRM/Alum**					
Appearance	Milky/turbid solution, no clumps	Milky/turbid solution, no clumps	Milky/turbid solution, no clumps	Milky/turbid solution, no clumps	Milky/turbid solution, no clumps
pH	7.0 – 7.8	7.45	Not tested	7.5	7.3
Adsorption to Alum	≥ 80%	> 99%	> 99%	> 99%	> 99%
p*17-CRM + K4S2-CRM/Alum					
Appearance	Milky/turbid solution, no clumps	Milky/turbid solution, no clumps	Milky/turbid solution, no clumps	Milky/turbid solution, no clumps	Milky/turbid solution, no clumps
pH	7.0–7.8	7.55	Not tested	7.6	7.4
Adsorption to Alum	≥ 80%	> 99%	> 99%	> 99%	> 99%

## Discussion

An extensive Strep A vaccine research program has led to the development of two lead vaccine candidates. Each vaccine candidate comprises a combination of the M protein peptide J8 or p*17, and the SpyCEP peptide, K4S2. The role of J8 and p*17 is to induce an antibody-mediated response to the major virulence factor of Strep A, the M protein. The peptides are designed so that the antibody-induced response has global utility in its ability to recognise multiple emm types. The role of K4S2 is to induce an antibody response to the virulence factor SpyCEP, broadening the protective spectrum of the vaccines to include Strep A CovR/S MT strains with upregulated SpyCEP. This is particularly relevant to Strep A invasive disease.

We have demonstrated that combining the M protein and SpyCEP peptides is an appropriate strategy for enhancing and broadening protection against Strep A infection. We previously reported that both J8-CRM/Alum and J8-CRM + K4S2-CRM/Alum were efficacious in protecting mice against CovR/S WT strain induced pyoderma and systemic infection, with more than a 95% reduction in skin and blood/spleen bacterial burden for both formulations. It was noted that protection afforded by K4S2-CRM alone was limited (20–30%). Likewise, J8-CRM/Alum alone was ineffectual in protection against CovR/S MT strain. These mice, had less than 40% reduction in both local and systemic bacterial burdens in comparison to the control mice. However, J8-CRM + K4S2-CRM/Alum was very effective in protecting against this MT strain and resulted in > 95% reduction in local and systemic bacterial burden. The data highlight the synergistic efficacy of K4S2 against CovR/S MT strains that upregulate SpyCEP expression^18^.

This paper provides additional supporting evidence for the combination vaccine approach of combining the M protein and SpyCEP peptides. Here we show that in outbred and inbred animal models of invasive infection, both vaccine candidates are efficacious against CovR/S WT and MT strains. To date, the data has been equally supportive of the protective efficacy and immunogenicity of both vaccine candidates, in animal models. The data supports the utility and safety of both vaccines. What is unknown is how either will perform in humans. This is something that will remain unknown until both are trialled and compared in human clinical trials.

The selection of a carrier protein, as a mechanism of enhancing the efficacy of the vaccines has been carefully considered. Early investigations were carried out with the peptides conjugated to the carrier protein, DT. DT was replaced with CRM, a chemically defined, genetically modified, non-toxic form of diphtheria toxin having a single amino acid substitution of glutamic acid. CRM has widespread use in licensed vaccines including Menveo, Hibtiter and Prevnar 13. We have previously reported on a study comparing CRM and DT conjugated to the Strep A peptides and determined that (1) DT or CRM conjugated vaccines induced comparable antigen specific antibodies and (2) prior exposure to DT had no detrimental effect on CRM conjugated vaccine efficacy^18^. Moving forward to clinical trials in humans, CRM is the carrier protein of choice.

We have previously reported on the binding capacity of antisera from mice vaccinated with DT to the streptococcal surface, as measured by immunofluorescence staining^12^. Binding was observed against both M1 and M6 strains. This cross-reactive binding was attributed to sequence homology between DT and Strep A and considered to be non-M type specific. We have also previously observed the limited binding capacity of CRM antibodies to CovR/S WT and MT Strep A^18^. In the whole cell ELISA, we again observe the limited binding capacity of CRM antibodies to Strep A. Given that CRM is the non-toxic analogue of diphtheria toxin, the binding of CRM reflects what we have seen before. The role of CRM is to induce a robust helper T cell-dependent response to the conjugated peptides, enabling the induction of specific antibodies. We argue that the additional recognition of Strep A by CRM antibodies has the effect of enhancing vaccine efficacy.

To be safe and in preparation for progression to clinical trials in humans, the vaccine should demonstrate no homology to human proteins or pose a risk of inducing autoimmunity, and demonstrate no toxicity in a suitable species.

The critical safety issue for a streptococcal vaccine is the potential for the vaccine to induce autoimmune pathology (RHD). ARF/RHD can occur after several untreated streptococcal infections and are believed to have an autoimmune aetiology^22^. Various antigens from the M protein have been shown to induce T-cells in mice that react with human cardiac myosin in vitro. These antigens have been identified in the amino terminal, the B-repeat and the C-repeat regions^23^.

The ability of the C-repeat region to induce cardiopathogenicity has been explored in rat valvulitis/carditis models. Madeleine Cunningham and colleagues have developed an animal model of rheumatic carditis in rats. A study in 2003 showed that immunization with a pool of 15 peptides spanning amino acids 337–492 from the C-repeat region (from where J8 [343–356] and p*17 [337–356] are located) could induce mononuclear cell infiltrates in the hearts of 4 of 5 Lewis rats^24^. More recently, rats were immunized with 25 peptides spanning the A-repeat, B-repeat and C-repeat regions of the M-protein. Peptides from the A-repeat region induced myocarditis, of similar magnitude as the entire protein; peptides from the B-repeat region induced mild carditis while peptides from the C-repeat region did not induce any carditis^25^.

A subsequent investigation of J8-DT in this same rat model demonstrated no cardiac lesions^13^. An additional study in 2016 from a different group tested the peptide, J14, which contains all 12 of the streptococcal amino acids present in J8 and 12 of the 20 amino acids present in p*17. This study also showed that the C-repeat region from where J8 and p*17 are located did not induce cardiac lesions^26^. Thus, the human and animal data point to an aetiology of RHD involving a response to sequences found in the A-repeat region of the M protein.

Homology searches were conducted between the individual peptides J8, p*17, K4S2 and the human proteome using the Universal Protein Resource database (UniProt)^27^. For J8, there were no matches for the full sequence and no alignments with greater than 85% sequence similarity (of any length). Sequence similarity ranged from 52 to 61%, all identified proteins had subcellular locations and there were no cardiac proteins identified. The data demonstrated that at the primary amino acid level J8 shows very little homology to the human proteins, specifically cardiac proteins. In a previous study, ProPred, RANKPEP and HLABIND algorithms failed to predict significant binding between the M protein specific regions of J8 and class II binding alleles. A single peptide was predicted to bind to HLA class I allele B2705. These data were supported by cellular proliferation assays demonstrating few peripheral blood mononuclear cells (PBMCs) from donors responding to J8. There was no correlation between proliferation to J8 and proliferation to host proteins^28^.

Based on the J8 data, and the lack of any toxicological findings in a study conducted independently by IIT Research Institute (Wisconsin), we previously undertook a human single-dose vaccine study of J8-DT/Alum. It was immunogenic and no adverse safety events were identified during the trial or the follow up period to 14 months^14^.

The homology search conducted between p*17 and the human proteome had similar results to that of J8. There were no matches for the full sequence and no alignments greater than 85% sequence similarity (of any length). Sequence similarity ranged from 50 to 80%, all identified proteins had subcellular locations. There were no cardiac proteins identified supporting previous in vitro studies demonstrating antibodies to p*17 did not bind to human myosin, tropomyosin or keratin^16^.

SpyCEP, from which K4S2 is derived, has not been implicated in the pathogenesis of rheumatic heart disease, and it is not expected that K4S2 will cross react with human tissues. Preclinical investigations of mouse K4S2 antisera to myosin and collagen have found no cross reactivity to these tissues (unpublished data). The homology search against the human proteome supported these findings. There were no cardiac proteins identified. There were no matches for the full sequence and no alignments above 85% sequence similarity (of any length). Sequence similarity ranged from 63 to 80%, 3 of the 4 identified proteins had subcellular locations, 1 protein was located in the extracellular region or secreted. K4S2 had a 69% (9/13) predicted sequence identity with residues 2021–2033 of Coagulation Factor V. Importantly, this identity falls outside of the critical pro-coagulant functioning C2 domain of Factor V (residues 2037-2196) where epitopes have been mapped for naturally occurring Factor V inhibitors and autoantibodies^29–31^.

Based on the above evidence, it was expected that it was safe to administer vaccines containing the J8, p*17 and K4S2 peptide conjugates.

Final conclusions from the toxicology study were that three (0.5 mL) intramuscular injections of 0.1 mg/dose J8-CRM + K4S2-CRM/Alum or 0.1 mg/dose p*17-CRM + K4S2-CRM/Alum to Sprague–Dawley rats over a period of 58 days followed by a 28 day recovery period was associated with increased white blood cell, lymphocyte and monocyte counts, increased adrenal gland weights, adrenocortical hypertrophy, and increased severity of granulomatous inflammation at the sites of injection and the associated inguinal lymph nodes. These changes were within the spectrum of expected findings post-vaccination and considered non-adverse.

Particular attention was paid to the presentation of any abnormal findings in organs or at sites commonly affected in Strep A disease. This included the heart or kidneys, as these are the principle sites of post-streptococcal sequelae pathology, and the brain and joints, afflicted in ARF. Of note, no adverse findings were reported in the kidneys, brain or the joints of any animals receiving vaccine.

A microscopic examination of the heart found no evidence of valvulitis, however, minimal cardiomyopathy was observed in 11 of 60 animals and mild cardiomyopathy in 1 animal. It should also be noted that the highest incidence of cardiomyopathy was observed in the J8-CRM + K4S2-CRM/Alum group. Previous studies with J8-DT/Alum in a rat model of cardiac valvulitis demonstrated that J8-DT did not induce valvulitis or myocarditis^13^. This further supports the finding that the cardiomyopathy observation in this study is not related to the administration of the vaccine, but related to spontaneous cardiomyopathy.

Local irritation (erythema and edema) was observed following dose 3. In the opinion of the study site veterinarian_,_ these types of reactions at the dose sites were expected, due to the dose volume being administered (0.25 mL per left and right thigh/dose). The total dose volume of 0.5 mL is the intended dose for the proposed clinical trial. Reviewing the clinical observations and the animals, the reactions were not considered to be due to the vaccines as the observations were in all groups. The overall conclusion was that a reduced dose volume would have likely resulted in a less severe reaction. It was also noted that the observation was of a local reaction rather than a systemic reaction. There was no change in body weight, food consumption or animal behaviour. In a non-toxicology related study, 6–8 week old male and female Sprague Dawley rats were subsequently intramuscularly administered 0.1 mL doses of 0.05 mg J8-CRM + K4S2-CRM/Alum or 0.05 mg p*17-CRM + K4S2-CRM/Alum at Days 1, 22 and 29. No edema was observed in the thighs of any rats (unpublished data). There was also no observed change to body weight, food consumption or animal behaviour.

Overall, based on the study outcomes, principally the development of desired immunological responses and the absence of clinical toxicity, the evidence supports both vaccines being safe for use in a phase 1 clinical trial in healthy humans.

## Methods

### Mouse immunization, skin challenge and sampling

Balb/c and SWISS mice (male and female, 4–6 weeks) were supplied by Animal Resource Centre (Perth, Western Australia). All protocols were approved by Griffith University’s Animal Ethics Committee in accordance with the National Health and Medical Research Council (NHMRC) of Australia guidelines. Methods were chosen to minimise pain and distress to the mice. Animals were observed daily by trained animal care staff. SWISS mice were immunized intramuscularly in the hind leg, with 0.025 mg of J8-CRM + K4S2-CRM formulated in aluminium hydroxide (Alhydrogel 2%, Alum) (Brenntag Biosector, Denmark) in 1:1 ratio (v/v), as previously described^17^. Balb/c mice were immunized with 0.025 mg of p*17-DT + K4S2-DT formulated in Alum. Control mice received saline with adjuvant alone. In all experiments, one day prior to immunization mice were bled via lateral tail vein and serum isolated. Serum samples were stored at − 20 °C until analysed by ELISA to determine murine Ag-specific IgG antibody titers as previously described^32^. Two–three weeks following final immunization, mice were challenged via the skin route of infection as previously described^10^. Animal passaged Strep A strains pNS1 (*emm* 100, CovR/S wild type) and 5448AP (*emm* 1, CovR/S mutant type) were used in this SWISS mice study. Animal passaged Strep A strain 5448AP (*emm* 1, CovR/S mutant type) was used in the Balb/c mice study. Post-challenge, the mice were monitored closely for their welfare. On days 3 and 6 post challenge, five mice were terminated, using CO_2_ inhalation, and blood and skin samples were collected for assessment of bacterial burden. To determine CFU in skin, the entire section of skin lesion was homogenised and tenfold serial dilutions were plated on blood agar plates. To assess systemic infection, tenfold serial dilution of blood samples were plated and CFU enumerated after the plates were incubated overnight at 37 °C.

### Strep A binding assay (whole cell ELISA)

Stationary phase cultures were prepared by inoculating Strep A into sterile Todd Hewitt Broth (THB) (Oxoid) supplemented with 1% neopeptone and 1% yeast and incubated stationary overnight at 37 °C. Next day, cultures were pooled and centrifuged to pellet Strep A cells. Supernatant decanted and cells resuspended in sterile DPBS (Gibco) and centrifuged at 4000xg for 10 min to wash the cells. DPBS was then decanted and the cell pellet was resuspended in DPBS and OD reading taken at 600 nm. Cell suspensions were then normalised to OD10 and aliquoted into smaller cell aliquots into safe lock Eppendorf tubes. Bacteria are heat killed at 65 °C for 60 min. Total protein concentration of the cell suspension was determined through BCA. Maxisorp microtitre plates (NUNC) were then coated at 0.2 mg/mL bacteria suspension in carbonate coating buffer and incubated at overnight at 4 °C. Plates are blocked with 0.2 mL of 5% skim milk/sterile PBS at 37 °C for 90 min. Plates are washed with PBS alone and controls and individual rat test sera applied (as serial dilution 1:2 starting at 1/100 to 1/819200), followed by HRP conjugated antibody against rat IgG (Biorad) at 37 °C for 90 min. Plates washed and detected with OPD substrate (Sigma) and read at 450 nm after 15 min incubation in the dark. Negative control serum was from naïve rats and a reference positive control was from rats immunized with conjugated peptides (J8-CRM-K4S2-CRM/Alum, p*17-CRM-K4S2-CRM/Alum or CRM/Alum). Test samples were considered positive when the mean value of the absorbance (OD at 450 nm), of the highest dilution (1:100) was > 3 standard deviations above the mean OD of the negative control.

### Toxicology study vaccine and control articles

Peptides conjugates J8-CRM, p*17-CRM and K4S2-CRM were manufactured by Auspep Clinical Peptides (Melbourne, Australia). Conjugates were > 95% pure and tested for endotoxin. The adjuvant, aluminium hydroxide (Alhydrogel 2%, Alum), was commercially sourced from Brenntag Biosector, Denmark. The J8-K4S2/Alum vaccine was formulated in Alum with 0.1 mg/ml J8-CRM and 0.1 mg/ml K4S2-CRM; the p*17-K4S2/Alum vaccine was formulated in Alum with 0.1 mg/ml p*17-CRM and 0.1 mg/ml K4S2-CRM to obtain a final total peptide conjugate concentration of 0.1 mg/0.5 ml dose, for each vaccine. The control article CRM/Alum was formulated in Alum with 0.130 mg/ml CRM_197_ (Pfenex) to obtain a final concentration of 0.065 mg/0.5 ml dose. This represents the approximate total concentration of CRM in each vaccine.

### Toxicology study

The toxicology study was conducted by Citoxlab, USA in compliance with the Food and Drug Administration Good Laboratory Practice Regulations as set out in Title 21 of the United States Code of Federal Regulations, Part 58. The study was designed with reference to the ICH M3(R2): Guidance on Non-Clinical Safety Studies for the Conduct of Human Clinical Trials and Marketing Authorization for Pharmaceuticals and the FDA Redbook 2000: General Guidelines for Designing and Conducting Toxicity Studies. With the exception of the manufacturing and characterization of the test and control articles and the immunogenicity analysis, all procedures were performed by the test facility.

### Animals

Male and female Sprague–Dawley rats were supplied by Charles River Laboratories, Inc. At the onset of dosing, the animals were 9.5 weeks old. The body weights ranged from 290.7 to 371.2 g and from 177.6 to 218.2 g for males and females, respectively. On arrival, all animals were subjected to a health assessment. Animals were group housed in an animal room where the environment was set to maintain a temperature of 20 to 26 °C; a relative humidity of 50 ± 20%; a light dark cycle of 12-h light/12-h dark, except during study protocol-designated procedures; and a minimum of 10 air changes per hour. All rats were given free and continuous access to food (Purina Mills Certified Rodent Diet 5002 in “meal” form; ad libitum) with the exception of fasting prior to scheduled clinical pathology collections and necropsy. All animals were given free and continuous access to tap water. An acclimation period of 15 days was allowed between receipt of the animals and the start of dosing to accustom the animals to the laboratory environment.

### Administration of vaccine doses

Twenty rats (10 male; 10 female) per group were administered 0.1 mg/0.5 mL of J8-CRM + K4S2-CRM/Alum, 0.1 mg/0.5 mL of p*17-CRM + K4S2-CRM/Alum or 0.065 mg/0.5 mL CRM/Alum at days 1, 22 and 43. The injection sites were shaved free of hair a day prior to each administration. The dose formulation was allowed to come to room temperature, and the 0.5 mL dose volume was administered as one 0.25 mL intramuscular injection to the right quadricep (thigh muscle) and one 0.25 mL intramuscular injection to the left quadricep.

### Assessment of repeat dose toxicity

Experimental endpoints were moribundity/mortality (at least once daily), clinical signs/physical examinations*,* inoculation site scoring for reactogenicity (erythema and edema), body weights*,* food consumption*,* ophthalmology, clinical pathology (clinical chemistry, hematology, coagulation, urinalysis), organ weights (adrenal glands, brain, epididymis, heart, kidney, liver and lungs), immunogenicity, gross necropsy observations and histopathology analyses. Microscopic examinations were performed on the aorta, heart, brain, eyes, gross lesions, injection sites, kidney, liver and lungs. All animals were bled prior to dosing and ten females and ten males of each group were bled prior to necropsy. Blood samples were collected with EDTA for hematological tests, or without anticoagulant for biochemical tests. Neutral buffered 10% formalin was used for fixation and preservation of tissues. Immunogenicity was performed at Griffith University and all other analysis were performed at Citoxlab, USA.

### Clinical pathology (clinical chemistry, coagulations, urinalysis and hematology)

Clinical chemistry parameters were sample appearance, creatinine, A/G ratio, globulin, alanine aminotransferase, glucose albumin, Phosphorus, alkaline phosphatase, potassium aspartate, aminotransferase, protein, bilirubin, sodium, calcium, triglycerides, chloride, urea, cholesterol and gamma-glutamyltransferase. Coagulation parameters were activated partial thromboplastin time and prothrombin time. Urinalysis parameters were bilirubin, blood, colour and appearance, glucose, ketones, leukocytes, pH, protein, specific gravity, urobilinogen, nitrites and microscopic examination of sediment. Hematology parameters measured were cell morphology, hematocrit, hemoglobin concentration, mean corpuscular volume, mean corpuscular hemoglobin, mean corpuscular hemoglobin concentration, platelet count, red blood cell count, red cell distribution width, reticulocyte counts, white blood cell count and white blood cell differential.

### Local irritation assessment

An evaluation of skin reactions for erythema/eschar and edema (scale 0–4) at the dosing site was performed prior to each day of dosing, and at approximately 3, 24, 48, and 72 h post-dose. Dermal observations were recorded using a modified Draize scoring scheme (Draize, 1959)^33^. Evaluations were performed on the animal’s right and left dose sites.

### Immunogenicity

Sera antibody titers to specific peptides were quantified using ELISA*.* Briefly, specific peptide was coated onto microtiter plates, controls and test sera applied (as serial dilution 1:2 starting at 1/100 to 1/3276800), followed by HRP conjugated antibody against rat IgG. Negative control serum was from rats immunized with Phosphate Buffered Saline and Alum and a reference positive control was from rats immunized with individual conjugated peptide (J8-CRM, p*17-CRM or K4S2-CRM). Test samples were considered positive when the mean value of the absorbance (OD at 450 nm), of the highest dilution (1:100) was 3 standard deviations above the mean OD of the negative control. For toxicity analysis, serum end point titer determined after three doses. For stability reporting, serum end point titer determined after one dose.

### Adsorption to alum

The efficiency of the adsorption of Alum in the vaccine was assessed by measuring the amount of the peptide conjugate that was not bound onto the adjuvant as described^18^. Briefly, vaccine samples were centrifuged and the supernatant collected in order to assess the amount of free peptide conjugate. The protein concentration in the supernatant was determined using a commercial micro-BCA estimation kit (Thermo Scientific). The total calculated amount of peptide conjugate present in the sample was used to calculate the percentage of adjuvant bound peptide conjugate. Adsorption is considered efficient if more than 80% of the total amount of peptide conjugate is bound onto adjuvant.

### Statistical analysis

Mean and standard deviations were calculated, as appropriate, for all quantitative data. As applicable, continuous group mean data that were examined statistically were evaluated for equality or homogeneity of variance using the Decision Tree statistical structure. The Decision Tree statistical structure includes analysis of variance (ANOVA), non-parametric analysis of variance, pairwise tests by the Dunnett’s Test for parametric and non-parametric data, simple t-tests, and the Bartlett’s Test (Dunnett, 1955) for homogeneity of variance. The data were analyzed for dose-related trends using the Williams Test (parametric data) or the Shirley Test (nonparametric data). Nonhomogeneous data were analyzed using a stepwise Dunnett’s Test (parametric data) or a modified Steel Test (nonparametric data). Frequency data (i.e., incidence data, etc.) that were examined statistically were evaluated using the Chi-Square and/or Fisher’s Exact Tests. Statistical tests were performed as two-sided tests with results taken as significant with probability (*p*) levels of < 0.05 or < 0.01. Statistical analysis was performed with SAS software. Antibody titer data are presented as geometric mean with geometric SD, with data analysis performed using GraphPad Prism version 8.1.2 for Windows, GraphPad Software, San Diego, California USA.

## Supplementary Information

Supplementary Information
