# Supplementary material for: Preclinical safety and immunogenicity of Streptococcus pyogenes (Strep A) peptide vaccines
Source: Sci Rep. 2021 Jan 8;11:127. doi: 10.1038/s41598-020-80508-6 (PMC7794325; doi:10.1038/s41598-020-80508-6)
Supplement: Supplementary file 1 — Supplementary Information [file 41598_2020_80508_MOESM1_ESM.pdf]

Preclinical Safety and Immunogenicity of *Streptococcus pyogenes* (Strep A) Peptide Vaccines

Simone Reynolds[#1*], Manisha Pandey[#1*], Jessica Dooley[1], Ainslie Calcutt[1], Michael Batzloff[1], Victoria Ozberk[1], Jamie-Lee Mills[1], Michael Good[1*]

# These authors contributed equally to this work.

* Corresponding authors: Simone Reynolds (simone.reynolds@griffith.edu.au); Manisha Pandey (m.pandey@griffith.edu.au); Michael Good (Michael.good@griffith.edu.au).

[1]Institute for Glycomics, Griffith University, Gold Coast, Australia.

Supplementary Information

Supplementary Table S1. Organs and tissues for histopathological examination.
Supplementary Table S2. Injection Site Histopathology.
Supplementary Table S3. Inguinal Lymph Node Histopathology.
Supplementary Table S4. Adrenal Gland Histopathology

Supplementary Table S1. Organs and tissues for histopathological examination

Adrenal Gland (2)	Rectum	Lung with large bronchi
Bone, femur (with articular surface of distal end)	Seminal vesicle	Lymph nodes (mandibular) (2)
Brain	Skin/subcutis	Mammary gland (females only)
Cervix uteri	Spleen	Optic nerve (2)
Duodenum	Testes	Oviduct
Esophagus	Thyroid with parathyroid	Parathyroid gland
Gross lesions	Trachea	Pituitary gland
Heart	Uterus	Prostate (males only)
Injection sites (Quadriceps femoris)	Aorta	Salivary gland (mandibular (2))
Jejunum	Bone marrow (sternum)	Sciatic nerve
Liver	Caecum	Spinal cord (cervical, thoracic and lumbar)
Lymph nodes (inguinal) (2)	Colon	Stomach
Lymph nodes (mesenteric)	Epididymis (2)	Thymus
Muscle (gastrocnemius)	Eye (2)	Tongue
Ovaries	Harderian gland (2)	Urinary bladder
Pancreas	Ileum	Vagina
Peyer's patch	Kidney (2)	Any tissue showing abnormal presentation upon necropsy
Physical identifier		

**Supplementary Table S2.** Injection Site Histopathology						
**Injection Site, right.** Inflammation, granulomatous						
	**Males (5 per group)**			**Females (5 per group)**		
**Day 57**	CRM	J8-K4S2	p*17-K4S2	CRM	J8-K4S2	p*17-K4S2
Minimal	1	0	1	0	0	0
Mild	4	3	4	2	1	2
Moderate	0	1	0	3	4	2
Marked	0	0	0	0	0	1
**Injection Site, right.** Inflammation, granulomatous						
	**Males (5 per group)**			**Females (5 per group)**		
**Day 86**	CRM	J8-K4S2	p*17-K4S2	CRM	J8-K4S2	p*17-K4S2
Minimal	0	0	0	0	0	0
Mild	5	5	2	5	1	4
Moderate	0	0	3	0	3	0
Marked	0	0	0	0	0	1
**Injection Site, left.** Inflammation, granulomatous						
	**Males (5 per group)**			**Females (5 per group)**		
**Day 57**	CRM	J8-K4S2	p*17-K4S2	CRM	J8-K4S2	p*17-K4S2
Minimal	1	0	0	2	0	1
Mild	3	1	2	2	5	2
Moderate	1	3	3	1	0	2
Marked	0	0	0	0	0	0
**Injection Site, left.** Inflammation, granulomatous						
	**Males (5 per group)**			**Females (5 per group)**		
**Day 86**	CRM	J8-K4S2	p*17-K4S2	CRM	J8-K4S2	p*17-K4S2
	No evidence			No evidence		

Numbers in table represent the number of animals with the finding.
CRM: CRM/Alum; J8-K4S2: J8-CRM+K4S2-CRM/Alum; p*17-K4S2; p*17-CRM+K4S2-CRM/Alum

Supplementary Table S3. Inguinal Lymph Node Histopathology

Inguinal lymph node, right. Inflammation, granulomatous

	**Males**			**Females**		
**Day 57**	CRM	J8-K4S2	p*17-K4S2	CRM	J8-K4S2	p*17-K4S2
**(No. examined)**	**2**	**3**	**4**	**4**	**5**	**4**
Minimal	0	0	0	1	0	0
Mild	0	1	2	1	1	2
Moderate	2	0	2	2	0	2
Marked	0	1	0	0	2	0

Inguinal lymph node, right. Inflammation, granulomatous

	**Males**			**Females**		
**Day 86**	CRM	J8-K4S2	p*17-K4S2	CRM	J8-K4S2	p*17-K4S2
	No evidence			No evidence		

Inguinal lymph node, left. Inflammation, granulomatous

	**Males**			**Females**		
**Day 57**	CRM	J8-K4S2	p*17-K4S2	CRM	J8-K4S2	p*17-K4S2
**(No. examined)**	**4**	**5**	**5**	**5**	**5**	**4**
Minimal	0	0	0	1	0	0
Mild	0	2	2	0	0	2
Moderate	3	2	3	2	2	1
Marked	1	0	0	1	2	1

Inguinal lymph node, left. Inflammation, granulomatous

	**Males**			**Females**		
**Day 86**	CRM	J8-K4S2	p*17-K4S2	CRM	J8-K4S2	p*17-K4S2
**(No. examined)**	**4**	**5**	**5**	**5**	**5**	**5**
Mild	1	2	1	1	1	1
Moderate	1	1	2	3	2	1
Marked	0	0	0	0	1	2

Numbers in table represent the number of animals with the finding.
CRM: CRM/Alum; J8-K4S2: J8-CRM+K4S2-CRM/Alum; p*17-K4S2; p*17-CRM+K4S2-CRM/Alum

Supplementary Table S4. Adrenal Gland Histopathology

Adrenal Gland. Hypertrophy, cortical

	**Males (5 per group)**		
**Day 57**	CRM	J8-K4S2	p*17-K4S2
Minimal	0	1	2
Mild	0	2	1

Numbers in table represent the number of animals with the finding.
CRM: CRM/Alum; J8-K4S2: J8-CRM+K4S2-CRM/Alum;
p*17-K4S2; p*17-CRM+K4S2-CRM/Alum
